# Supplementary material for: LIANA+ provides an all-in-one framework for cell–cell communication inference
Source: Nat Cell Biol. 2024 Sep 2;26(9):1613–22. doi: 10.1038/s41556-024-01469-w (PMC11392821; doi:10.1038/s41556-024-01469-w)
Supplement: Supplementary file 1 — Supplementary Notes 1–10 and references. [file 41556_2024_1469_MOESM1_ESM.pdf]

nature cell biology

Supplementary information https://doi.org/10.1038/s41556-024-01469-w

LIANA+ provides an all-in-one framework for cell–cell communication inference

In the format provided by the authors and unedited

Supplementary Information

Supplementary Note 1. LIANA+ enables the scalable estimation of CCC from single-cell and spatial (multi-)omics data.

To benchmark the efficiency of components in LIANA+, we generated simulated datasets ranging from 1,000 to 100,000 observations (cells). We drew random counts from a Poisson distribution with an assumed gene sparsity of ~90% across 10 cell types, to generate a representation that fits the properties of UMI-based transcriptomics data [1]. For dissociated methods, we generated a simulated resource of 2,000 interactions, while for spatially-weighted local scores, we used 1,000 - reflecting the typically lower readouts captured by sequencing-based spatial datasets. We assigned x and y coordinates as random integers between 0 and 5000, and using those we calculated spatial proximities considering the 10 nearest neighbours and a Gaussian kernel with a bandwidth of 150 and cutoff at 0.1.

For dissociated methods, we ran all methods with default parameters, with the number of permutations being set to 1,000 for permutation-based methods and parallel jobs to 4. For local metrics, we also used the default parameters of LIANA+, utilising all CPU threads.

For multi-view learning from spatial data, we used cell type proportions from a myocardial infarction Visium slide (ACH0013) as targets (intra-view), while extra views were generated randomly with the approach described above. Each extra view had 500 randomly generated genes and the same number of spots as the intra view.

For multi-view factor analysis of intercellular communication programmes from single-cell data, we generated 20 samples using the approach above. Then to generate a different range of views (cell type pairs), we iterated over a range of distinct cell type numbers, starting at 4 (16 cell type pairs) and ending at 20 (400 cell type pairs).

We ran each evaluation five times using a personal laptop (Dell XPS15 2019 model), with an Intel Processor i7-9750H and 16GB of RAM.

Our results showed that LIANA+ scales well across ranges of observations in single-cell (**Supp. Figure S1A**) and spatial data (**Supp. Figure S1B**). Moreover, it remains efficient when learning across a range of spatial views (**Supp. Figure S1C**) as well as with increasing numbers of cell type pairs (views) when using multi-view factor analysis (MOFA+) [2] (**Supp. Figure S1D**). Similarly, the standard workflows of LIANA+, available at the time of writing, are all executable in under 10 minutes on a standard laptop.

Workflow running time and RAM statistics (**Supp. Table 1**) were calculated using the start-to-finish vignettes of LIANA+ (v1.0.5), available at https://liana-py.readthedocs.io.

Supplementary Note 2. LIANA+'s Prior Knowledge Component

All components of LIANA+ rely on existing biological knowledge. As such, LIANA+ draws from OmniPath's rich database of ligand-receptor resources [3], providing access to 15 different resources, along with a consensus resource. Moreover, via OmniPath, we obtain a signed and directed intracellular protein-protein interaction as well as CollecTRI's generalistic transcription factor regulons [4].

To increase the flexibility of our CCC workflows, the knowledge in LIANA+ was further expanded by leveraging BioCypher knowledge [5]. For instance, we use and access the MetalinksDB graph [6] - a comprehensive and customisable resource of metabolite-protein interactions, additionally incorporating annotations such as tissues, pathways, and diseases. To provide a light-weight interface to MetalinksDB, we access it as a standard relational database, making use of the inbuilt sqlite3 Python library (https://docs.python.org/3/library/sqlite3.html).

An overview of the prior knowledge used by LIANA+ is shown here: https://liana-py.readthedocs.io/en/latest/notebooks/prior_knowledge.html.

Supplementary Note 3. LIANA+ implements bivariate metrics to study co-localisations from spatially resolved data.

Common notations:

$x \in \mathbb{R}^n$ and $y \in \mathbb{R}^n$ are vectors of two variables for n spots (or cells),

$\bar{x}$ and $\bar{y}$ are the mean of the variable values,

$w \in \mathbb{R}^{n \times n}$ is a spatial proximity weight matrix indicating the degree of spatial association between spots i and j.

Inspired by scHOT [7], we implemented local weighted variants of common similarity metrics, such as Pearson and Spearman correlation:

$$\text{wPearson}\rho_i = \frac{\sum w_{ij} \sum (w_{ij} x_j y_j) - \sum (w_{ij} x_j) \sum (w_{ij} y_j)}{\sqrt{(\sum w_{ij} \sum (w_{ij} x_j^2) - \sum (w_{ij} x_j)^2)(\sum w_{ij} \sum (w_{ij} y_j^2) - \sum (w_{ij} y_j)^2)}}$$

$$\text{wSpearman}\rho_i = \frac{\sum w_{ij} \sum (w_{ij} r_{x_j} r_{y_j}) - \sum (w_{ij} r_{x_j}) \sum (w_{ij} r_{y_j})}{\sqrt{(\sum w_{ij} \sum (w_{ij} r_{x_j}^2) - \sum (w_{ij} r_{x_j})^2)(\sum w_{ij} \sum (w_{ij} r_{y_j}^2) - \sum (w_{ij} r_{y_j})^2)}},$$

where the summation is performed over j and r_{xj} , r_{yj} are ranks of x and y for spot j.
A second masked version of Spearman correlation, as proposed and the default approach in scHOT, was also implemented; where we consider r_{xj} , r_{yj} only for spots with non-zero w.

Moreover, we provide weighted Jaccard and cosine similarity metrics:

$$\text{wJaccard}_i = \frac{\sum_{j=1}^{n} w_{ij} \min(x_j, y_j)}{\sum_{j=1}^{n} w_{ij} \max(x_j, y_j)}$$

where: max/min operations are carried out along all observations of x and y, following each being binarised by setting values > 0 to 1, thus signifying the presence or absence of a readout.

$$\text{wCosine}_i = \frac{\sum_{j=1}^{n} w_{ij} x_j y_j}{\sqrt{\sum_{j=1}^{n} w_{ij} x_j^2} \sqrt{\sum_{j=1}^{n} w_{ij} y_j^2}}$$

We also implement a simple spatially-weighted product (as in NICHES [8]) and a max-normalised product as a scale-invariant counterpart:

$$\text{wProduct}_i = \left(\sum_{j=1}^{n} w_{ij} x_j \right) \left(\sum_{j=1}^{n} w_{ij} y_j \right)$$

$$\text{wNormProduct}_i = \left(\frac{\sum_{j=1}^{n} w_{ij} x_j}{\max(|x'|)} \right) \left(\frac{\sum_{j=1}^{n} w_{ij} y_j}{\max(|y'|)} \right)$$

where $x' \in \mathbb{R}^n$ and $y' \in \mathbb{R}^n$ are the spatially-weighted vectors of x and y respectively

We noticed that the weighted product formulation corresponds to the local L-statistic - another bivariate spatial association metric [9,10] when the x and y variables are z-transformed.

We further adapted bivariate Global and local Moran's R, extensions of Moran's I [11], from SpatialDM [12]; both of which are measures of spatial co-occurrence.

Local Moran's R is defined as:

$$\text{Local}R_i = (x_i - \bar{x}) \sum_{j=1}^{n} w_{ij}(y_j - \bar{y}) + (y_i - \bar{y}) \sum_{j=1}^{n} w_{ij}(x_j - \bar{x})$$

In contrast to Local Moran's R, Global Moran's R infers the co-clustering of two variables globally, and is defined as:

$$\text{Global R} = \frac{\sum_i^n \sum_j^n w_{ij}(x_i - \bar{x})(y_j - \bar{y})}{\sqrt{\sum_i^n (x_i - \bar{x})^2} \sqrt{\sum_i^n (y_i - \bar{y})^2}}$$

Along with the local metrics, we enable the user to obtain the categories of interactions. If spatially-weighted variables are non-negative (e.g. gene expression) then they first are z-transformed. Then for each spot i, we categorise interactions according to the signs of the x and y variables involved in the interaction as positive (1), negative (-1), or neither (0):

$$\text{Let } s_x = \text{sign}\left(\sum_{j=1}^{n} x_j w_{xj} \right) \text{ and } s_y = \text{sign}\left(\sum_{j=1}^{n} y_j w_{yj} \right)$$

$$\text{Category}_{\text{i}}(s_x, s_y) = \begin{cases} 1 & \text{if } s_x > 0 \text{ and } s_y > 0 \\ -1 & \text{if } (s_x > 0 \text{ and } s_y < 0) \text{ or } (s_x < 0 \text{ and } s_y > 0) \\ 0 & \text{if } s_x < 0 \text{ and } s_y < 0, \text{ or either } s_x = 0 \text{ or } s_y = 0 \end{cases}$$

This calculation of the local categories is independent of the computation of local scores. However, we also enable setting the scores of non-positive interactions to 0 and their permutation-based P-values to 1 via a ***mask_negatives*** parameter.

Supplementary Note 4. Spot calling Evaluation of the Spatially-weighted Local Metrics implemented in LIANA+.

First, we evaluated the ability of the spatially-weighted local metrics to classify malignant and non-malignant spots in four breast cancer slides [13] (Methods). All scoring functions did well at classifying malignancy (AUROC > 0.9; weighted F1 > 0.85; **Extended Data Fig. 2A-B**). Spatially-informed Jaccard, cosine, and the products had slightly higher AUROCs (average ≈ 0.95) and F1 scores (average > 0.88) than other methods across the slides, generally followed by weighted Pearson and Spearman correlations, masked Spearman correlation, and finally bivariate Moran's R (**Extended Data Fig. 2A-B**).

Second, using 28 spatial transcriptomics slides from myogenic, ischemic, and fibrotic heart tissue upon myocardial infarction [14], we evaluated the ability of local ligand-receptor scores to recover cell type proportions (Methods). We noted that the spatially-weighted products, along with cosine and Jaccard similarities, had slightly higher predictive performance in ischemic (R^2 > 0.32) and fibrotic tissues (R^2 > 0.27) than the rest of the metrics, while Moran's R did best in the myogenic slide (R^2 ≈ 0.13) (**Extended Data Fig. 2C**); with similar results also observed in terms of Root Mean Squared Error (**Extended Data Fig. 2D**).

In summary, all spatially-informed local scores in LIANA+ performed well at predicting malignancy and cell type specificity, suggesting they preserved the biological signal captured by gene expression. Yet, our results suggested that spatially-weighted products, Jaccard, and cosine similarity performed on average best in both the regression and classification tasks, albeit marginally better than other methods. From these well-performing local metrics, we chose cosine similarity as LIANA+'s default, also used throughout the manuscript, since it's easily interpretable (being bound between -1 and +1) and does not require the data to be binarised.

Nevertheless, the other scoring metrics are well suited for other tasks. For example, the spatially-weighted Jaccard index is well suited for categorical or binary data. Spearman correlation is more appropriate to infer ordinal or ranked relationships between variables. Similarly, metrics such as Moran's R, or the simple and normalised products, are useful in cases in which there is no overlap between the two variables (e.g. one-hot encodings), as they spatially weigh the variables prior to calculating any relationship between them. Thus, the choice of metric should take into consideration the data and task at hand.

Supplementary Note 5. Evaluation of ligand-receptor methods using spatially resolved, transcriptome-wide single-cell RNA-Seq data

We used the recent Slide-tags technology [15] to compare ligand-receptor methods' performance [16]. In contrast to our previous work [16], and that of others [17,18], which relied on integrating matched spatial and single-cell RNA-Seq datasets, this technology enables the inference of ligand-receptor interaction and generation of assumed truth using the same data. Moreover, rather than using solely the colocalization of cell types alone [16], here we additionally take into account the colocalization of ligand-receptor interactions (Methods).

Using this setting we compared CellPhoneDB [19], CellChat [20], Connectome [21], NATMI [22], SingleCellSignalR [23], scSeqComm [24], log2FC, Geometric Mean, and LIANA's consensus (Rank Aggregate) across five slide-tag datasets [15]. First, we generated AUROC curves and saw that the individual scoring functions of most methods performed marginally better than random (AUROC > 0.5), with the inter-dataset variance being too high to suggest which method works best (**Fig. 4A**).

Next, we evaluated the effectiveness of the false positive filtering thresholds suggested by each method (if available; Methods). To account for the unequal class distributions, we used Balanced Accuracy to compare the methods. We again saw that the methods' performances were closely aligned with random (**Fig. 4B**).

Finally, as done by a previous benchmark [17], we computed F1 scores, but saw a notable correlation with the number of interactions retained by each method (Spearman's rho = 0.765). Moreover, the different filtering processes across methods result in each predicting a distinct set of interactions, thereby further limiting the effectiveness of this metric. As such, we opted to use a normalised F1 score which considered the random outcome anticipated for each method (Methods). Using these normalised F1 scores, we again saw that the methods and their individual scores (when capped at the 95th quantile) performed only slightly better than random (**Fig. 4C&D**). Moreover, our benchmark highlighted that scoring functions that focus on the magnitude of interactions (e.g. LIANA's magnitude rank and ligand-receptor means or products), provide superior performance than functions that reflect interaction specificity across cell type pairs [25] (**Fig. 4A&C**).

Taken together, we saw that methods generally exceeded random performance, albeit marginally. Yet, the large variance across datasets and methods limited our ability to suggest the method that works best. Moreover, such a setting can only highlight which interactions are unlikely to occur but remains limited as expression co-localisation does not necessitate interaction. Instead, our evaluation suggests that regardless of method, ligand-receptor interaction inference, uninformed of spatial location, is anticipated to result in high false positive rates. Thus, our results highlight the need for supporting information [16].

Supplementary Note 6. Combinations of ligand-receptor scores with Multi-view factor analysis or Tensor-cell2cell evaluated using Sample Label Classification.

In contrast to our hypothesis-testing approach (see **Fig. 5L**), and those proposed by other methods [12,26,27], unsupervised, or higher-order dimensionality reduction approaches, can simultaneously model CCC events across samples and cell types [28]. As a consequence, rather than considering each interaction on its own, these approaches capture and summarise coordinated interactions into intercellular programmes. Moreover, these approaches inform us about the most relevant cell types or interactions that separate the samples according to some condition, along with summaries of model performance, such as the amount of variance explained or reconstruction error.

Recently, we aligned Tensor-cell2cell and LIANA to enable the user-friendly inference of context-specific CCC patterns with any method or resource from the latter [29]. Here, we showcase the use of an alternative unsupervised approach leveraging multi-view factorisation via the MOFA+ framework [2].

This approach inherits the efficiency and flexibility of multi-views to enable factor analysis of CCC interactions by modelling pairs of cell groups as views. As such, it enables feature flexibility across the cell-type pairs and provides ligand-receptor importances per cell-type pair and factor. Moreover, it allows the simultaneous decomposition of the input data and alignment with additional covariates [30], similar to a recent CCC decomposition approach [31].

To evaluate the ability of both Tensor-cell2cell and multi-view factorisation in identifying intercellular programmes that distinguish samples from different conditions, we set up a classification task (Methods). We used five public cross-conditional atlases from the human heart, lung, and brain (**Supp. Table 4**), combining each of the ligand-receptor methods in LIANA+ with both dimensionality reductions (**Extended Data Fig. 5A**).

In each dataset, we inferred interactions independently for each sample using the scoring functions from each ligand-receptor method in LIANA+, utilising magnitude-based scores if provided (**Supp. Table 3**). Then, we used multi-view factorisation and Tensor-cell2cell to decompose the ligand-receptor output, obtained per sample (**Extended Data Fig. 5A**). Using a binary classification setup, we calculated AUROC and F1 for each method-factorisation combination to examine well each classified condition (**Extended Data Fig. 5A**; Methods).

We saw that all combinations performed better than random in most datasets (**Extended Data Fig. 5B**), with log2FC (AUROC=0.89) and the expression average of ligand-receptor means (CellPhoneDB; AUROC=0.87) performing best when combined with multi-view factorisation; while the rest of the methods had an AUROC ≈ 0.8, except CellChat (AUROC=0.62); though this does not necessarily reflect the performance of the CellChat. When combined with Tensor-cell2cell, methods showed slightly higher variance; with the

product (AUROC=0.84) and Rank Aggregate (LIANA's consensus; AUROC=0.82) having the highest AUROCs, while the rest of the methods' AUROCs ranged between 0.69 and 0.76, except SingleCellSignalIR (AUROC=0.6). We also saw similar results when using weighted F1 (**Extended Data Fig. 5C)**. Moreover, methods showed on average higher AUROCs when combined with MOFA+ than Tensor-cell2cell. With the exceptions of CellChat which performed better when combined with Tensor-cell2cell, and the Carraro dataset on which methods combined with Tensor-cell2cell showed better performance. This potentially reflects an intrinsic difference in the regularizations used between the two approaches. Specifically, the multi-view factorisation uses view-, factor- and feature-wise regularisation [2], while the non-negative tensor component analysis (PARAFAC) used by Tensor-cell2cell, which can be thought of as a higher-order extension of NMF [28], does not.

Taken together, in addition to performing on average better at capturing condition-relevant variance, our proposed approach, using multi-view factorisation to extract intercellular programmes, provides interaction importance per cell-type pair and is highly efficient, without the need for GPU acceleration (**Extended Data Fig. 1D; Supp. Table 1**). Nevertheless, our results suggest that both Tensor-cell2cell and multi-view factorisation consistently capture condition-relevant intercellular programmes, regardless of the ligand-receptor method used.

Supplementary Note 7. LIANA+ leverages a rich knowledge base to link CCC events to intracellular signalling.

Leveraging knowledge from OmniPath [3] and BioCypher [5,6] (**Fig. 2B**), LIANA+ provides access to both protein-mediated and metabolite-mediated [6] ligand-receptor interactions, which can be further annotated and contextualised according to pathways, disease, or locations of interest (**Fig. 2C**), among others. Such annotations can also be used to perform downstream enrichment analysis on the output of any implemented CCC methods. For example, in the myocardial infarction analysis, we used enrichment analysis [32] to identify active pathways [33] associated with ligand-receptor loadings from ischemic and fibrotic heart regions [14].

In addition, LIANA+ can infer signalling networks to link identified CCC events to downstream intracellular signalling pathways and transcription factors. In contrast to existing network methods used in the CCC field [34,35], our approach considers the direction of deregulation for nodes of interest. Specifically, it evaluates the sign of deregulation (activation or inhibition) in ligands, receptors, and transcription factors, alongside the signalling pathways linking them. To do so, we incorporate knowledge of protein-protein interactions (activating or inhibiting), ligand-receptor interactions, and transcription factors with their targets (**Fig. 2B**; Methods). Then a network-optimisation approach [36,37] (**Fig. 2D**) is used to identify causal paths that connect deregulated CCC events (input nodes) with active transcription factors (output nodes) (**Fig. 2E**; Methods). It is further noteworthy that the approach implemented here is not bound specifically to linking protein-mediated CCC interactions to TFs, but can be flexibly applied to find links between any set of nodes, including such between metabolites and downstream signalling [38].

As such, LIANA+ provides a comprehensive suite to decipher and interpret intracellular signalling related to CCC.

Supplementary Note 8. ***Prediction integration with LIANA+ improves prediction reliability across datasets.***

To demonstrate the unique contributions of each LIANA+ component, we illustrated the steps in the analysis shown in **Fig. 5**. This showed that LIANA+ substantially reduces the prediction space from a potential >1M interactions down to a reliable and interpretable set of mechanistic inter- and intracellular hypotheses in specific cell types of interest (**Extended Data Fig. 7A**).

Moreover, we quantified the reliability gained by integrating complementary LIANA+ components using an unsupervised clustering task, utilising another five public single-cell heart failure atlases (**Supp. Table 5**). In this task, we evaluated how well a small number of predictions can distinguish, in an unsupervised manner, between myeloid cell samples from heart failure patients versus healthy controls. This analysis revealed an increase in Silhouette scores and a performance significantly above a random baseline when we combined the predictions from single-cell and spatial LIANA+ components (Step 3) (z-score > 1.645; **Extended Data Fig. 7B**). Moreover, the final analysis step, which combined ligand-receptor and cell-type predictions from both single-cell and spatial data with downstream signalling events, resulted in the highest Adjusted Rand Index (ARI) across all steps (**Extended Data Fig. 7C**). In this step, LIANA+ predictions had an ARI higher than random predictions across all datasets, with four of those being close to or significantly better than random (P-value < 0.05; **Extended Data Fig. 7D**).

These results highlight LIANA+'s capacity to integrate complex single-cell and spatial datasets, and hence generate interpretable and robust condition-relevant predictions.

Supplementary Note 9. LIANA+ deals with spatial modalities with different spatial coordinate systems.

To enable multi-view learning [39] across any number of views, regardless of their dimensions, spatial connectivity weights (calculated using a radial basis kernel) are estimated according to a reference coordinate system. Let $X \in \mathbb{R}^{n \times d1}$ and $Y \in \mathbb{R}^{m \times d2}$ represent the data matrices from two distinct modalities, but from the same tissue section. Here, n and m represent the number of observations in each modality, while $d1$ and $d2$ denote the number of variables specific to each modality. The spatial connectivity weights are captured in a matrix $W \in \mathbb{R}^{n \times m}$, which quantifies the spatial proximity between each observation in Y to each observation in X. Then the alignment of these modalities is achieved through a simple matrix multiplication: $Y' = WY$. Here, Y' represents the transformed version of Y, with a new shape of $Y \in \mathbb{R}^{n \times d2}$, aligning Y with the observation space of X while maintaining the feature dimensions of the modality.

Similarly, to enable the calculation of local metrics that necessitate a calculation of a relationship between features from the modalities prior to spatial weighting or transformation, we use linear grid interpolation as implemented in scipy [40]. This method interpolates one matrix to the reference coordinate space - typically the modality with a lower number of observations.

Programmatically, the calculation of spatial connectivities in LIANA+ mirrors Squidpy's ***spatial_neighbors*** function, and thus spatial connectivities can be easily replaced with Squidpy's neighbourhood graphs [41]. Similarly, for operations such as image alignment, segmentation, or coordinate transformations LIANA+ relies on other general-purpose spatial frameworks [42–44].

Supplementary Note 10. LIANA+ implements a sign-coherent subnetwork search to link intra- and intercellular signalling.

In LIANA+, we implemented a modified version of CARNIVAL [38] that takes four distinct inputs: (1) a prior knowledge graph (PKN) of signed protein-protein interactions, where nodes are proteins and edges are activating or inhibitory interactions; continuous and signed (2) starting (input) nodes and (3) end (output) node values, with negative values indicating downregulation and positive values indicating upregulation. In addition, we take (4) values for the rest of the nodes in the graph [0, 1] (e.g. gene expression proportions), with higher values incurring less penalty than genes with lower values when the gene is included in the inferred network. Then, a subnetwork, optimised for sparsity, is extracted from the PKN which connects the input (starting) nodes to the output (end) nodes, taking into account both the directionality and sign of interaction.

The resulting inferred network is a directed acyclic graph that connects the (2) input nodes to the (3) end nodes (e.g. receptor to transcription factors), including the values for each edge and node of the graph indicating if the node is upregulated (+1), or downregulated (-1). A node n_c in the graph can be upregulated only if there is at least one selected parent node n_p such that n_p is upregulated and there is an activating edge between n_p and n_c, or n_p is inhibited and there is an inhibitory edge between n_p and n_c. Similarly, a node n_c can be downregulated if there is a parent node n_p downregulated with an activating edge between n_p and n_c, or if a parent node n_p is upregulated and there is an inhibitory edge between n_p and n_c.

These rules are encoded using linear constraints and continuous/binary variables to define a Mixed integer linear programming problem, which is a particular type of combinatorial problem with linear constraints. The optimization problem is defined as:

$$\arg\min_{\mathbf{x}^+,\mathbf{x}^-} L(\mathbf{x}^+,\mathbf{x}^-) = \sum_{i:v_i>0}(1-x_i^+)v_i + \sum_{i:v_i<0}(1-x_i^-)|v_i| + \sum_i \lambda_i(x_i^+ + x_i^-)$$

where x^+ is a vector of binary variables for each node in the PKN indicating whether the node i is upregulated ($x_i^+ = 1$) or not; x^- is a vector of binary variables for each node in the PKN indicating whether node i in the PKN is downregulated ($x_i^- = 1$) or not; v is a vector of values for measured nodes (input nodes and output nodes), where positive values are upregulated species and negative values are downregulated species. For example, v can be estimated as fold change, t-statistic, or any other score indicating a difference in activity in a protein in the PKN between two conditions.

Here, we additionally introduce λ - a vector of penalties to penalise the inclusion of protein nodes in the resulting inferred network, according to (4) node weights w [0, 1] in the (1)

PKN. Specifically, we use λ to discretise highly-expressed from lowly-expressed genes, thus accounting for the differences in drop-out rates across cell types in single-cell data.

We set λ to $\text{penalty}_{\text{max}}$ (1 as default) and $\text{penalty}_{\text{min}}$ (0.01 as default) according to a threshold (0.1) by default:

$$\lambda_i = \begin{cases} \text{penalty}_{\text{max}} & \text{if } w_i \leq \text{threshold} \\ \text{penalty}_{\text{min}} & \text{if } w_i > \text{threshold} \end{cases}$$

Linear constraints impose conditions on the variables of the optimisation problem. For example, a node cannot be upregulated and downregulated at the same time ($x^{+} + x^{-} \leq 1$). The problem includes other variables and linear constraints to guarantee that the final networks' valid solutions are acyclic networks and that the rules explained before are respected. Additional information about the formulation can be found in Liu et al. (2019) [36].

References

1. Pan, Y. *et al.* The Poisson distribution model fits UMI-based single-cell RNA-sequencing data. *BMC Bioinformatics* **24**, 256 (2023).
2. Argelaguet, R. *et al.* MOFA+: a statistical framework for comprehensive integration of multi-modal single-cell data. *Genome Biol.* **21**, 111 (2020).
3. Türei, D. *et al.* Integrated intra‐ and intercellular signaling knowledge for multicellular omics analysis. *Mol. Syst. Biol.* **17**, (2021).
4. Müller-Dott, S. *et al.* Expanding the coverage of regulons from high-confidence prior knowledge for accurate estimation of transcription factor activities. *Nucleic Acids Res.* **51**, 10934–10949 (2023).
5. Lobentanzer, S. *et al.* Democratizing knowledge representation with BioCypher. *Nat. Biotechnol.* **41**, 1056–1059 (2023).
6. Farr, E. B. *et al.* MetalinksDB: a flexible and contextualizable resource of metabolite-protein interactions. *BioRxiv* (2023) doi:10.1101/2023.12.30.573715.
7. Ghazanfar, S. *et al.* Investigating higher-order interactions in single-cell data with scHOT. *Nat. Methods* **17**, 799–806 (2020).
8. Raredon, M. S. B. *et al.* Comprehensive visualization of cell-cell interactions in single-cell and spatial transcriptomics with NICHES. *Bioinformatics* **39**, (2023).
9. Lee, S.-I. Developing a bivariate spatial association measure: An integration of Pearson’s r and Moran’s I. *J. Geogr. Syst.* **3**, 369–385 (2001).
10. Wang, L., Liu, C., Gao, Y., Zhang, X. H.-F. & Liu, Z. Unravelling spatial gene associations with SEAGAL: a Python package for spatial transcriptomics data analysis and visualization. *Bioinformatics* **39**, (2023).
11. Anselin, L. A local indicator of multivariate spatial association: extending geary’s *c*. *Geogr. Anal.* **51**, 133–150 (2019).
12. Li, Z., Wang, T., Liu, P. & Huang, Y. SpatialDM for rapid identification of spatially co-expressed ligand-receptor and revealing cell-cell communication patterns. *Nat.*

Commun. **14**, 3995 (2023).

13. Wu, S. Z. *et al.* A single-cell and spatially resolved atlas of human breast cancers. *Nature Genetics* (2021).
14. Kuppe, C. *et al.* Spatial multi-omic map of human myocardial infarction. *Nature* **608**, 766–777 (2022).
15. Russell, A. J. C. *et al.* Slide-tags enables single-nucleus barcoding for multimodal spatial genomics. *Nature* **625**, 101–109 (2024).
16. Dimitrov, D. *et al.* Comparison of methods and resources for cell-cell communication inference from single-cell RNA-Seq data. *Nat. Commun.* **13**, 3224 (2022).
17. Liu, Z., Sun, D. & Wang, C. Evaluation of cell-cell interaction methods by integrating single-cell RNA sequencing data with spatial information. *Genome Biol.* **23**, 218 (2022).
18. Luo, J., Deng, M., Zhang, X. & Sun, X. ESICCC as a systematic computational framework for evaluation, selection, and integration of cell-cell communication inference methods. *Genome Res.* (2023) doi:10.1101/gr.278001.123.
19. Efremova, M., Vento-Tormo, M., Teichmann, S. A. & Vento-Tormo, R. CellPhoneDB: inferring cell-cell communication from combined expression of multi-subunit ligand-receptor complexes. *Nat. Protoc.* **15**, 1484–1506 (2020).
20. Jin, S. *et al.* Inference and analysis of cell-cell communication using CellChat. *Nat. Commun.* **12**, 1088 (2021).
21. Raredon, M. S. B. *et al.* Computation and visualization of cell-cell signaling topologies in single-cell systems data using Connectome. *Sci. Rep.* **12**, 4187 (2022).
22. Hou, R., Denisenko, E., Ong, H. T., Ramilowski, J. A. & Forrest, A. R. R. Predicting cell-to-cell communication networks using NATMI. *Nat. Commun.* **11**, 5011 (2020).
23. Cabello-Aguilar, S. *et al.* SingleCellSignalR: inference of intercellular networks from single-cell transcriptomics. *Nucleic Acids Res.* **48**, e55 (2020).
24. Baruzzo, G., Cesaro, G. & Di Camillo, B. Identify, quantify and characterize cellular communication from single-cell RNA sequencing data with scSeqComm. *Bioinformatics* **38**, 1920–1929 (2022).

25. Luecken, M. D. *et al.* Defining and benchmarking open problems in single-cell analysis. *Res. Sq.* (2024) doi:10.21203/rs.3.rs-4181617/v1.
26. Lagger, C. *et al.* scAgeCom: a murine atlas of age-related changes in intercellular communication inferred with the package scDiffCom. *BioRxiv* (2021) doi:10.1101/2021.08.13.456238.
27. Browaeys, R. *et al.* MultiNicheNet: a flexible framework for differential cell-cell communication analysis from multi-sample multi-condition single-cell transcriptomics data. *BioRxiv* (2023) doi:10.1101/2023.06.13.544751.
28. Armingol, E. *et al.* Context-aware deconvolution of cell-cell communication with Tensor-cell2cell. *Nat. Commun.* **13**, 3665 (2022).
29. Baghdassarian, H. M., Dimitrov, D., Armingol, E., Saez-Rodriguez, J. & Lewis, N. E. Combining LIANA and Tensor-cell2cell to decipher cell-cell communication across multiple samples. *Cell Rep. Methods* 100758 (2024) doi:10.1016/j.crmeth.2024.100758.
30. Velten, B. *et al.* Identifying temporal and spatial patterns of variation from multimodal data using MEFISTO. *Nat. Methods* **19**, 179–186 (2022).
31. Dai, Q., Epstein, M. P. & Yang, J. STACCato: Supervised Tensor Analysis tool for studying Cell-cell Communication using scRNA-seq data across multiple samples and conditions. *BioRxiv* (2023) doi:10.1101/2023.12.15.571918.
32. Badia-I-Mompel, P. *et al.* decoupleR: ensemble of computational methods to infer biological activities from omics data. *Bioinformatics Advances* **2**, vbac016 (2022).
33. Schubert, M. *et al.* Perturbation-response genes reveal signaling footprints in cancer gene expression. *Nat. Commun.* **9**, 20 (2018).
34. Hu, Y., Peng, T., Gao, L. & Tan, K. CytoTalk: De novo construction of signal transduction networks using single-cell transcriptomic data. *Sci. Adv.* **7**, (2021).
35. Browaeys, R., Saelens, W. & Saeys, Y. NicheNet: modeling intercellular communication by linking ligands to target genes. *Nat. Methods* **17**, 159–162 (2020).
36. Liu, A. *et al.* From expression footprints to causal pathways: contextualizing large signaling networks with CARNIVAL. *NPJ Syst. Biol. Appl.* **5**, 40 (2019).

37. saezlab/corneto: CORNETO: an optimization library for modeling biological network inference problems. https://github.com/saezlab/corneto.
38. Dugourd, A. *et al.* Causal integration of multi-omics data with prior knowledge to generate mechanistic hypotheses. *Mol. Syst. Biol.* **17**, e9730 (2021).
39. Tanevski, J., Flores, R. O. R., Gabor, A., Schapiro, D. & Saez-Rodriguez, J. Explainable multiview framework for dissecting spatial relationships from highly multiplexed data. *Genome Biol.* **23**, 97 (2022).
40. Virtanen, P. *et al.* SciPy 1.0: fundamental algorithms for scientific computing in Python. *Nat. Methods* **17**, 261–272 (2020).
41. Palla, G. *et al.* Squidpy: a scalable framework for spatial omics analysis. *Nat. Methods* **19**, 171–178 (2022).
42. Chen, J. G. *et al.* Giotto Suite: a multi-scale and technology-agnostic spatial multi-omics analysis ecosystem. *BioRxiv* (2023) doi:10.1101/2023.11.26.568752.
43. Pham, D. *et al.* Robust mapping of spatiotemporal trajectories and cell-cell interactions in healthy and diseased tissues. *Nat. Commun.* **14**, 7739 (2023).
44. Marconato, L. *et al.* SpatialData: an open and universal data framework for spatial omics. *Nat. Methods* (2024) doi:10.1038/s41592-024-02212-x.
